# Supplementary material for: CoMix: comparing mixing patterns in the Belgian population during and after lockdown
Source: Sci Rep. 2020 Dec 14;10:21885. doi: 10.1038/s41598-020-78540-7 (PMC7736856; doi:10.1038/s41598-020-78540-7)
Supplement: Supplementary file 1 — Supplementary Information 1. Supporting Information (SI) [file 41598_2020_78540_MOESM1_ESM.pdf]

Covid 19 behaviour and contact survey

Démographie

Quel âge avez-vous (en année)?

Quel est votre sexe ?

- Homme
- Femme
- Autre
- Je préfère ne pas répondre

Où habitez-vous?

- Province:
- Postal Code:
- City:

Q3. Quel est votre statut professionnel actuel ?
Veuillez sélectionner une seule réponse

1. Employé(e) à temps plein (34 heures ou plus)
2. Employé(e) à temps partiel (moins de 34 heures)
3. Indépendant(e)
4. Au chômage, en recherche de travail
5. Au chômage, pas en recherche de travail
6. Personne au foyer
7. Retraité(e)
8. Étudiant(e) / élève
9. Malade de longue durée, ou personne en situation de handicap

Are you the one in your household who has the highest income? [person with the largest income from employment, pensions, state benefits, investments or other sources]

1. Yes
2. Yes, together with another household member
3. No

What is your highest level of education attained?

1. Without a diploma or primary education
2. General lower secondary education (first 3 years completed)
3. Technical, artistic or professional lower secondary education (first 3 years completed)
4. General upper secondary education (6 years completed)
5. Technical or artistic upper secondary education (6 years)
6. Professional upper secondary (6 years)
7. Higher education: graduat, candidature, bachelor
8. University education: bachelor's degree, post-graduate, master's degree
9. Complementary master
10. 0 Doctorate

What is your occupation?

What is the profession that you last exercised?

What is the highest education level of the main earner?

What is the occupation of the main earner?

What is the profession that the main earner last exercised?

Êtes-vous actuellement enceinte ?

- Oui
- Non
- Je préfère ne pas répondre

Quel est votre revenu familial NET TOTAL (c'est-à-dire le montant après déduction fiscale), en incluant chaque source de revenu ? Ici, la famille inclue toutes personnes marié(e)s, en union civil, ou uni(e)s par des liens de naissance ou d'adoption et vivant à la même adresse.

Please include all your income sources : salaries, scholarships, pension and Social Security benefits, dividends from shares, income from rental properties, child support and alimony etc.

- 0€ - 549€
- 550€ - 999€
- 1 000€ - 1 299€
- 1 300€ - 1 499€
- 1 500€ - 1 699€
- 1 700€ - 1 899€

- 1 900€ - 2 199€
- 2 200€ - 2 499€
- 2 500€ - 2 799€
- 0 2 800€ - 3 199€
- 1 3 200€ - 3 699€
- 2 3 700€ - 4 499€
- 3 4 500€ - 5 499€
- 4 5 500€ - 7 999€
- 5 8 000€ ou plus
- 6 Je préfère ne pas répondre

Sans vous inclure, combien de personnes vivent dans votre foyer ? Ici, la définition de foyer inclut toutes les personnes vivant à la même adresse que vous, avec qui vous partagez une cuisine.
Aucune
1
2
3
4
5
6
7
8
9
10
11 ou plus

Laquelle des propositions suivantes décrit le mieux votre foyer?

2. Deux adultes ou plus de familles différentes
3. Couple avec un ou plusieurs enfant(s) à charge
4. Couple avec un ou plusieurs enfant(s), dont aucun n'est à charge
5. Couple sans enfant
6. Parent seul avec un ou plusieurs enfant(s) à charge.
7. Parent seul avec un ou plusieurs enfant(s), dont aucun n'est à charge
8. Foyer composé de deux familles ou plus
9. Autre

Veuillez s'il vous plaît renseigner le nom ou les initiales de chaque personne vivant dans votre foyer.
Notez que ces noms ou initiales sont uniquement utilisés afin de faciliter le remplissage de cette enquête. Les noms individuels ne sont pas visibles pour quiconque en dehors de cette enquête. Si vous le préférez, vous pouvez utiliser des initiales.

*ASK THE FOLLOWING QUESTIONS FOR EACH [*NAME] *FILLED*
Quel est l'âge de [NAME] (en années) ?

1. Moins de 1
2. 1-4
3. 5-9
4. 10-14
5. 15-19
6. 20-24
7. 25-34
8. 35-44
9. 45-54
10. 55-64
11. 65-69
12. 70-74
13. 75-79
14. 80-84
15. 85+
16. Je ne sais pas
17. Je préfère ne pas répondre

Quel est le sexe de [NAME] ?

- Homme
- Femme
- Autre
- Je préfère ne pas répondre
- Je ne sais pas

Quel est le statut professionnel actuel de [NAME] ?

- Employé(e) à temps plein (34 heures ou plus)
- Employé(e) à temps partiel (moins de 34 heures)
- Indépendant(e)
- Au chômage, en recherche de travail
- Au chômage, pas en recherche de travail
- Personne au foyer
- Retraité(e)
- Étudiant(e) / élève
- Malade de longue durée, ou en situation de handicap

Q26. Est-ce que [NAME] est un(e) étudiant(e) ou un(e) élève dans l'un des établissements suivants ?

1. Crèche ou école maternelle
   2. École primaire
   8. École secondaire
   4. Enseignement supérieur, p. ex. université

5. Aucune des options ci-dessus
6. Je ne sais pas
7. Je préfère ne pas répondre

[NAME] est-elle actuellement enceinte ?
Oui
Non
Je ne sais pas
Je préfère ne pas répondre

FLU
Q28. Êtes-vous ou un autre membre de votre foyer est-il dans un groupe à risque, donnant droit à un vaccin gratuit contre la grippe saisonnière annuelle ?
Les groupes à risque sont composés des personnes souffrant d'une affection chronique, de nature pulmonaire, cardiaque, hépatique, neurologique ou rénale, de diabète, d'immunosuppression (due à une maladie ou à un traitement), d'asplénie ou dysfonctionnement de la rate, d'obésité de classe III (IMC ≥ 40), et des femmes enceintes.
*ASK THE FOLLOWING QUESTIONS FOR EACH [*NAME] *FILLED*

ROWS:
Vous
Nom 1
Nom 2
etc.

COLUMNS:
1. Oui
2. Non
3. Je ne sais pas
4. Je préfère ne pas répondre

Symptômes

Avez-vous, ou quelqu'un dans votre foyer a-t-il eu un ou plusieurs de ces symptômes durant les sept derniers jours ?
*ASK THE FOLLOWING QUESTIONS FOR EACH [*NAME] *FILLED*
Please tick all that apply

ROWS:
Vous
Nom 1
Nom 2
etc.

COLUMNS:
1. Fièvre ou température élevée
2. Une toux qui a duré au moins quelques heures
3. Essoufflement
4. Courbatures (p. ex. douleurs au dos, à la nuque, aux épaules ou aux articulations)
5. Nez bouché
6. Mal de gorge
7. Fatigue inhabituelle
8. Aucun de ces symptômes
9. Je ne sais pas
10. Je préfère ne pas répondre

ASK ALL WHO HAVE SUFFERED ANY SYMPTOMS (1 OR 2 AT SYMPTOM)
Après l'arrivée des symptômes, avez-vous, ou quelqu'un dans votre foyer a-t-il effectué l'une des actions suivantes ?

ROWS:
Vous
Nom 1
Nom 2
etc.

COLUMNS:
1. Appeler le numéro vert 0800 14 689 ou visiter le site gouvernemental info-coronavirus.
2. Appeler un médecin généraliste
3. Aller au cabinet d'un médecin généraliste
4. NOT APPLICABLE
5. Aller à un service d'urgences
6. Aller dans un centre de dépistage autre que les propositions précédentes
7 Être admis dans un hôpital
8. Je ne sais pas
9. Aucune des options précitées
10. Je préfère ne pas répondre

Vous avez indiqué que [IF FOR SELF AT SERVICE – 'vous' OR IF FOR [NAME] AT SERVICE – 'NAME'] [avez/a] réalisé l'action suivante : [INSERT SERVICE FROM SERVICE]. Quand [avez-vous / a-t-il/elle] fait cela ? Si vous ne connaissez pas la date exacte, veuillez s'il vous plaît renseigner une

estimation.
1. INSERT DATE *INSERT DATE USE DATE FORMAT DD/MM*
2. Je ne sais pas
3. Je préfère ne pas répondre

Est-ce que quelqu'un dans votre foyer, ou vous-même, avez déjà été testés pour le coronavirus (Covid-19) ?

- ○ Oui
- ○ Non

ASK IF TEST IS YES
ASK FOR SELF AND EACH PERSON NAMED
Qui dans votre foyer a été testé pour le coronavirus (Covid-19) ?
ASK FOR SELF AND EACH PERSON NAMED
NOTE FOR SCRIPTER – PLEASE SCRIPT AS GRID

ROWS:
Vous
Nom 1
Nom 2
etc.

COLUMNS:
1. Testé, et le résultat du test était positif pour le coronavirus
2. Testé, et le résultat du test était négatif pour le coronavirus
3. Oui, et le résultat du test n'a pas encore été reçu
4. Non testé
5. Je ne sais pas
6. Je préfère ne pas répondre

ASK FOR SELF AND EACH PERSON NAMED

À votre connaissance, pensez-vous que vous-même ou quelqu'un d'autre de votre foyer avez été en contact direct avec une personne atteinte du coronavirus (Covid-19) au cours des sept derniers jours, ou connaissez-vous un proche atteint du coronavirus (Covid-19) ?
Veuillez sélectionner une réponse par ligne.

ASK FOR SELF AND EACH PERSON NAMED

ROWS:
Vous
Nom 1
Nom 2
etc.

COLUMNS:
1. Oui, actuellement infecté
2. Oui, décédé
3. Oui, guéri
4. Non
5. Je ne sais pas
6. Je préfère ne pas répondre

Attitudes
À quel point êtes-vous en accord ou en désaccord avec les phrases suivantes ?

1.Tout à fait d'accord
2. D'accord
3. Ni en désaccord, ni d'accord
4. Pas d'accord
5. Pas du tout d'accord
6. Je ne sais pas

1. Le coronavirus serait une maladie grave pour moi
2. Il est probable que j'attrape le coronavirus
3. Si je ne suis pas les recommandations du gouvernement, il est possible que je transmette le coronavirus à une personne vulnérable.

EFFECT

À quel point pensez-vous que les méthodes suivantes soient efficaces pour empêcher la propagation du coronavirus ?

1. Très efficace
2. Assez efficace
3. Peu efficace
4. Pas du tout efficace
5. Je ne sais pas

1. Réduire le nombre de personnes que vous rencontrez en face à face
2. Rester chez vous pendant 7 jours en cas de symptômes légers tels qu'une toux légère
3. Rester chez vous pendant 7 jours en cas de symptômes plus sévères tels qu'une toux sévère ou une température élevée
4. Éviter les lieux hautement fréquentés
5. Rester chez vous pendant 14 jours si une autre personne de votre foyer développe des symptômes légers tels qu'une toux légère
6. Rester chez vous pendant 14 jours si une autre personne de votre foyer développe des symptômes sévères tels qu'une toux sévère ou une température élevée
7. Fermer les écoles
8. Fermer les bars, les restaurants, les cinémas etc.
9. Interdire l'usage des transports en commun
10. Interdire les vols internationaux vers la Belgique
11. Interdire les vols domestiques en Belgique

À quel point êtes-vous confiant(e) que si vous le vouliez, vous pourriez… ?

1.Très confiant(e)
2. Assez confiant(e)
3. Peu confiant(e)
4. Pas du tout confiant(e)
5. Je ne sais pas

1. Réduire le nombre de personnes que vous rencontrez
2. Rester chez vous pendant 7 jours en cas de symptômes légers tels qu'une toux légère
3. Rester chez vous pendant 7 jours en cas de symptômes plus sévères tels qu'une toux sévère ou une température élevée
4.. Éviter les lieux hautement fréquentés
5. Rester chez vous pendant 14 jours si une autre personne de votre foyer développe des symptômes légers tels qu'une toux légère
6. Rester chez vous pendant 14 jours si une autre personne de votre foyer développe des symptômes sévères tels qu'une toux sévère ou une température élevée
7. Ne pas utiliser de transports en commun

À quel point êtes-vous en accord ou en désaccord avec les phrases suivantes ?

1.Tout à fait d'accord
2. D'accord
3. Ni en désaccord, ni d'accord
4. Pas d'accord

5. Pas du tout d'accord
6. Je ne sais pas

1. Les personnes avec lesquelles je travaille attendent de moi que je continue à travailler, même lorsque je suis malade
2. Si je ne pouvais pas travailler à cause du coronavirus, je serais quand même payé(e)
3. Si je devais m'isoler pendant 7 jours, quelqu'un d'autre serait capable de s'occuper de mes enfants
4. Si je devais m'isoler pendant 7 jours, d'autres personnes en dehors de mon foyer seraient affectées de manière négative
5. J'ai assez de nourriture et de provisions pour tenir 7 jours, si je devais m'isoler

Comportements
Il vous a peut-être été demandé ou vous avez peut-être décidé de participer à différentes mesures contre le coronavirus (covid-19).
Par rapport aux sept derniers jours, veuillez s'il vous plaît sélectionner la réponse appropriée pour chacune des mesures listées ci-dessous.

Durant les sept derniers jours, [IF FOR SELF – vous a-t-on demandé] [IF FOR NAME 1/2 ETC AT NAME – a-t-on demandé à NAME] de…
ASK FOR SELF AND EACH PERSON NAMED

ROWS:
1. [Vous/Se] mettre en quarantaine
La quarantaine est l'acte de rester chez soi pendant 14 jours suite à une exposition potentielle à un cas infecté. Si vous êtes en quarantaine, vous pouvez quitter votre domicile, mais devez limiter vos déplacements.
2. [Vous/Se] mettre en isolement
L'isolement est l'acte de se distancer des autres personnes qui ne sont pas infectées pendant 7 jours, y compris des potentiels membres du foyer. Vous pouvez être en isolement à votre domicile, ou dans un établissement de santé.
3. [Vous/Se] mettre en télétravail ou limiter [votre/son] temps passé sur [votre/son] lieu de travail (covid-19)
4. Limiter [votre/son] temps passé [à l'université] [ONLY SHOW THIS OPTION FOR THOSE CODING 8 AT EMP01 OR 4 AT QHH ATTEND] OR [à la crèche ou l'école maternelle] OR [à l'école primaire] OR [à l'école secondaire] à cause du coronavirus (covid-19)

COLUMNS:
1.Oui
2. Non
3. Ne s'applique pas
4. Je ne sais pas
5. Je préfère ne pas répondre

ASK FOR SELF AND EACH PERSON NAMED
Durant les sept derniers jours, est-ce que…

ROWS:
1. [Votre/Son] lieu de travail a été fermé pour au moins un jour

2. [[Votre/Son] université] OR [[votre/sa] crèche ou école maternelle] OR [[votre/son] école primaire] OR [[votre/son] [école secondaire] a été fermé(e) pour au moins un jour

COLUMNS:
1. Oui
2. Non
3. Ne s'applique pas
4. Je ne sais pas
5. Je préfère ne pas répondre

ASK FOR SELF AND EACH PERSON NAMED
Durant les sept derniers jours, [IF FOR SELF – avez-vous] [IF FOR NAME 1/2 ETC AT NAME est-ce que NAME a] …

ROWS:
1. Été mis(e) en quarantaine pendant au moins un jour
2. Été mis(e) en isolation pendant au moins un jour
3. Évité de [vous/se] rendre sur [votre/son] lieu de travail pendant au moins un jour
4. Évité de vous/se rendre [à l'université] OR [à la crèche ou l'école maternelle] OR [à l'école primaire] OR [à l'école secondaire] pendant au moins un jour

COLUMNS:
1. Oui
2. Non
3. Ne s'applique pas
4. Je ne sais pas
5. Je préfère ne pas répondre

ASK FOR EACH QUARANTINED PERSON
Vous avez répondu que [vous avez/NAME a] été mis(e) en quarantaine pendant au moins un jour. À quelle date [avez-vous/NAME a t'il/elle] commencé cette quarantaine ?
1. INSERT DATE
2. Je ne sais pas
3. Je préfère ne pas répondre

ASK FOR EACH QUARANTINED PERSON
Et à quelle date [avez-vous/NAME a] terminé cette quarantaine ?
Veuillez s'il vous plaît utiliser le format « JJ/MM »

1. INSERT DATE
2. [Je suis/ Il/elle] est toujours en quarantaine
3. Je ne sais pas
4, Je préfère ne pas répondre

ASK FOR EACH QUARANTINED PERSON
Vous avez dit que [vous avez/NAME a] été mis(e) en isolation pendant au moins un jour. A quelle date [avez-vous/NAME a] commencé cette isolation ?
Veuillez s'il vous plaît utiliser le format « JJ/MM »

1. INSERT DATE
2. Je ne sais pas
3. Je préfère ne pas répondre

ASK FOR EACH QUARANTINED PERSON
Et à quelle date [avez-vous/NAME a] terminé cette isolation ?
Veuillez s'il vous plaît utiliser le format « JJ/MM »

1. INSERT DATE
2. [Je suis/ Il/elle] est toujours en isolation
3. Je ne sais pas
4. Je préfère ne pas répondre

Vous avez répondu que [votre/...] [[.../le] lieu de travail] OR [[…/l'] université]OR [[…/l'] crèche ou école maternelle]OR [[…/l'] école primaire] OR [[…/l'] école secondaire] […/de NAME] a été fermé(e) pendant au moins un jour durant ces sept derniers jours. Veuillez s'il vous plaît indiquer la date à partir de laquelle ce lieu a été fermé.

Veuillez s'il vous plaît utiliser le format « JJ/MM »
1. INSERT DATE
2. Je ne sais pas
3. Je préfère ne pas répondre

Et à quelle date ce lieu a-t-il été ouvert de nouveau ?
Veuillez s'il vous plaît utiliser le format « JJ/MM »

INSERT DATE
Ce lieu est toujours fermé
Je ne sais pas
Je préfère ne pas répondre

Vous avez répondu que [vous ne vous êtes/NAME ne s'est] pas rendu(e) [au travail] OR [à l'université] OR [à la crèche ou l'école maternelle]OR [à l'école primaire] OR [à l'école secondaire] , alors que ce lieu était encore ouvert. Durant les sept derniers jours, veuillez s'il vous plait indiquer les jours pendant lesquels [vous ne vous êtes /NAME ne s'est] pas rendu(e) [au travail] OR [à l'université] OR [à la crèche ou l'école maternelle]OR [à l'école primaire] OR [à l'école secondaire] . Veuillez seulement indiquer les jours où [vous vous seriez/NAME se serait] normalement rendu(e) sur ce lieu.

1 - 7. SHOW DATES FOR LAST SEVEN DAYS
8. Je ne sais pas
9. Je préfère ne pas répondre

Quelle était la raison principale pour laquelle vous ne vous êtes pas rendu [au travail] OR [à l'université] [à la crèche ou l'école maternelle] OR [à l'école primaire] OR [l'école secondaire] ? Veuillez cocher toutes les réponses qui s'appliquent

1. En isolation pendant 7 jours car vous aviez des symptômes légers qui pouvaient correspondre au coronavirus
2. En quarantaine pendant 14 jours car une autre personne de votre foyer avait des symptômes qui pouvaient correspondre au coronavirus, ou car vous aviez été en contact avec un cas confirmé de coronavirus
3. Quelqu'un dans votre foyer (vous inclus(e)) avait une maladie autre que le coronavirus
4. Vous vous occupiez d'une personne ne faisant pas partie de votre foyer, et qui est un cas confirmé de coronavirus (COVID-19)
5. Vous vous occupiez d'une personne ne faisant pas partie de votre foyer, et qui n'est pas un cas confirmé de coronavirus (COVID-19)
6. Au moins un enfant dans votre foyer est chez vous à cause de la fermeture des écoles
7. Autre

Vous avez répondu que [vous ne vous êtes/NAME ne s'est] pas rendu(e) sur [votre/son] lieu de travail pendant au moins un jour. Est-ce que cela a eu un impact négatif sur le revenu de votre foyer ?

1. Non, j'ai pu faire du télétravail
2. Non, j'ai pu prendre un congé payé exceptionnel « pour soignant »
3. Non, mais j'ai dû prendre au moins un jour de congé payé
4. Non, car j'ai été entièrement dédommagé(e) par mon employeur
5. Non, car j'ai été entièrement dédommagé(e) par le gouvernement
6. Oui, mais j'ai été partiellement dédommagé(e) par mon employeur
7. Oui, mais j'ai été partiellement dédommagé(e) par le gouvernement
8. Oui, et je n'ai reçu aucune compensation pour mon revenu perdu
9. Autre (veuillez préciser)
10. Je ne sais pas
11. Je préfère ne pas répondre

Vous avez répondu que [la crèche ou l’école maternelle]OR [l’école primaire] OR [l’école secondaire] de [NAME] a été fermé(e) pendant au moins un jour à cause du coronavirus (covid-19). Lorsque cela est arrivé, qui a surveillé l’/les enfant(s) ?
Please tick all that apply

1. Un parent, qui est au chômage
2. Un parent, qui était en télétravail
3. Un parent, qui travaille à temps partiel
4. Un parent, qui a pris un congé payé
5. Un parent, qui a pris un congé payé exceptionnel « pour soignant »
6. Un parent, qui a pris un congé non-payé
7. Un frère ou une sœur
8. Un/des grand(s)-parent(s)
9. Un/une baby-sitter, assistant(e) maternelle, au pair ou nounou (payé(e))
10. Un/une baby-sitter, assistant(e) maternelle, au pair ou nounou (non-payé(e))
11. Un voisin, ami, oncle ou tante
12. Ce n’était pas nécessaire
13. Autre (veuillez préciser)
14. Quelqu’un de l’école, car mon enfant était éligible à la garde d’enfant de l’école

Vous avez répondu que [NAME] ne s’était pas rendu(e) [à la crèche ou l’école maternelle]OR [à l’école primaire] OR [à l’école secondaire] pendant au moins un jour à cause du coronavirus (covid-19). Lorsque cela est arrivé, qui a surveillé l’/les enfant(s) ?

1. Un parent, qui est au chômage
2. Un parent, qui était en télétravail
3. Un parent, qui travaille à temps partiel
4. Un parent, qui a pris un congé payé
5. Un parent, qui a pris un congé payé exceptionnel « pour soignant »
6. Un parent, qui a pris un congé non-payé
7. Un frère ou une sœur
8. Un/des grand(s)-parent(s)
9. Un/une baby-sitter, assistant(e) maternelle, au pair ou nounou (payé(e))
10. Un/une baby-sitter, assistant(e) maternelle, au pair ou nounou (non-payé(e))
11. Un voisin, ami, oncle ou tante
12. Ce n’était pas nécessaire
13. Autre (veuillez préciser)
14. Quelqu’un de l’école, car mon enfant était éligible à la garde d’enfant de l’école

Vous êtes-vous rendu(e), ou aviez-vous l'intention de vous rendre dans l'un ou plusieurs des lieux suivant durant les sept derniers jours ?

ROWS:

1. Pub, bar ou café
2. Restaurant
3. Cinéma
4. Concert
5. Supermarché, supérette ou marché
6. Un évènement sportif (en tant que spectateur), par exemple un match de football
7. Un évènement sportif (en tant que participant), par exemple un entraînement de tennis
8. Rassemblement religieux

9. Un lieu en intérieur, où plus de 100 personnes étaient présentes
10. Un lieu en extérieur, où plus de 100 personnes étaient présentes

COLUMNS:

1, Oui, je me suis rendu(e) sur ce lieu, ou j'ai participé à cet évènement
2. J'avais l'intention de me rendre sur ce lieu, mais cela a été annulé à cause de l'épidémie de coronavirus (covid-19)
3. J'avais l'intention de me rendre sur ce lieu, mais j'ai choisi de ne pas m'y rendre à cause de l'épidémie de coronavirus (covid-19)
4. J'avais l'intention de me rendre sur ce lieu, mais j'ai/cela a été annulé pour des raisons indépendantes de l'épidémie de coronavirus (covid-19)
5. Non, je ne me suis pas rendu(e) sur ce lieu, ou je n'ai pas participé à cet évènement

Vous avez répondu que vous [IF CODE 1 AT VISIT – vous êtes rendu(e), IF CODES 2-4 AT VISIT – aviez l'intention de vous rendre] [à INSERT EVENT OR LOCATION] [IF CODES 2-4 AT VISIT ENTER SECOND PART OF STATEMENT] mais que cela a été annulé à cause du coronavirus / mais que vous avez choisi de ne pas vous y rendre à cause du coronavirus / mais que vous avez ou que cela a été annulé pour des raisons indépendantes du coronavirus]. Combien de fois cela s'est-il produit durant les sept derniers jours ?
INSERT NUMBER OF TIMES
Je ne sais pas

Mesures préventives individuelles
Avez-vous porté un masque hier ?

1 .Oui
2. Non

Pendant combien de temps avez-vous porté un masque au total ?
Veuillez renseigner une durée totale approximative
INSERT NUMBER OF HOURS
INSERT NUMBER OF MINUTES

Ou avez-vous porté votre masque ?
Please tick all that apply
1. Partout en dehors de mon domicile
2. En marchant dans la rue
3. En faisant du vélo
4. Dans les transports publics
5. Dans les supermarchés/magasins
6. Au cinéma/bar/restaurant
7. Chez vous
8. Au travail/à l'école primaire/ à l'école secondaire/à l'université
9. Autre (veuillez préciser)

Combien de fois vous êtes-vous lavé les mains avec du savon durant ces trois dernières heures ?
INSERT NUMBER OF TIMES

Combien de fois avez-vous utilisé du désinfectant pour les mains durant ces trois dernières heures ?
INSERT NUMBER OF TIMES

Avez-vous voyagé dans des transports publics hier ?
1. Non
2. Train/métro
3. Bus/tram
4. Taxi, Uber, ou application de Voiture de Transport avec Chauffeur (VTC) similaire
5. Avion

Et combien de temps environ avez-vous passé dans [INSERT FROM Q59] au total ?

INSERT NUMBER OF HOURS
INSERT NUMBER OF MINUTES

Sondage de contact

INTRO SCREEN
Nous allons maintenant vous demander de vous rappeler des personnes avec lesquelles vous avez eu un contact direct hier, entre 5h du matin hier et 5h du matin aujourd'hui.

Ces questions sont volontaires, mais également très importantes pour nous aider à comprendre la propagation du COVID-19 et l'impact de différentes mesures de santé publique. Les résultats publiés ne permettront pas de vous identifier ou d'identifier tout membre de votre foyer.

Nous sommes uniquement intéressés par les contacts directs, c'est-à-dire des **personnes que vous avez rencontrées en face à face**, avec lesquelles vous avez échangé au moins quelques mots, ou avec lesquelles vous avez eu un contact physique (p . ex. étreinte, s'embrasser, sport de contact).
Si vous avez uniquement parlé à une personne par téléphone ou internet, celle-ci ne devrait pas être incluse dans cette section.

Avec lesquelles des personnes suivantes avez-vous eu un contact direct, entre 5h du matin hier et 5h du matin aujourd'hui, en face à face ?

ROWS:
INSERT ALL NAMES FROM QNAME

COLUMNS:
1. Oui
2. Non

Et avec quelles **autres personnes n'appartenant pas à votre foyer** avez-vous eu un contact direct en face à face, entre 5h du matin hier et 5h du matin aujourd'hui ? Cela pourrait inclure des amis, de la famille, des collègues de travail, ou des personnes avec lesquelles vous avez parlé dans un magasin etc.

Veuillez indiquer le nom de chaque personne ci-dessous. Notez que ce nom sera uniquement utilisé afin de faciliter le remplissage de cette enquête, veuillez donc choisir un nom facile à mémoriser. Vos réponses individuelles ne seront partagées avec personne hors du cadre de l'enquête.

Nous sommes uniquement intéressés par les personnes que vous avez rencontrées en face à face, ***avec lesquelles vous avez échangé au moins quelques mots, ou avec lesquelles vous avez eu un contact physique*** *(p. ex. poignée de main, étreinte, sport de contact).*

Il est plus simple d'énumérer les noms par ordre chronologique, p. ex. après avoir pris le petit-déjeuner à la maison, je suis allé(e) au travail et j'ai parlé à Jack, Déborah et deux clients. En rentrant chez moi, j'ai discuté avec l'employé de la station-service (donnez un nom comme « le vendeur »). etc. Quand je suis rentré(e) à la maison, j'ai réceptionné un colis auprès du livreur, et j'ai parlé à mon amie, Fatima, dans mon jardin. Etc.

Veuillez ne pas vous compter vous, toute personne que vous avez indiquée comme appartenant à votre foyer ou toute personne avec qui vous avez parlé uniquement au téléphone ou sur Internet.

You have already indicated contact with the following household members, add additional contacts in the textboxes below:

VEUILLEZ ÉNUMÉRER TOUS LES AUTRES CONTACTS QUE VOUS AVEZ HORS DE VOTRE FOYER, PAR EXEMPLE, J, D, CLIENT 1, CLIENT 2, VENDEUR, LIVREUR, F

Personne/personne d'autre

CONTACTAGE
Q66. Quel est l’âge de [INSERT NAME]’S en années ? Si vous n’en êtes pas sûr(e), veuillez fournir une estimation. Veuillez sélectionner une seule réponse.

1. Moins de 1
2. 1-4
3. 5-9
4. 10-14
5. 15-19
6. 20-24
7. 25-34
8. 35-44
9. 45-54
10. 55-64
11. 65-69
12. 70-74
13. 75-79
14. 80-84
15. 85+
16. Je ne sais pas
17. Je préfère ne pas répondre
18. Cette personne est moi

Pourriez-vous s’il vous plait renseigner une estimation de l’âge de [INSERT NAME] ?

[INSERT AGE FROM] à [INSERT AGE TO]

Je préfère ne pas répondre

Est-ce que [NAME] est …

- ○ Un homme
- ○ Une femme
- ○ Autre
- ○ Je préfère ne pas répondre

Quelle est la relation de [NAME] par rapport à vous ?

1. Il/elle est un(e) membre de ma famille qui n'est pas dans mon foyer
2. Il/elle est un(e) collègue
3. Il/elle est une personne avec laquelle je vais à l'école ou à l'université
4. Il/elle est un(e) ami(e)
5. Autre
6. Je préfère ne pas répondre

À quelle fréquence avez-vous des contacts directs avec [NAME] en temps normal (c.-à-d. avant l'épidémie) ?
Un contact direct correspond à **une rencontre en face à face avec cette personne**, et lorsque vous échangez au moins quelques mots, ou lorsque vous avez un contact physique (p. ex. poignée de main, étreinte, s'embrasser, sport de contact).
Veuillez ne pas inclure les occasions où vous échangez avec cette personne par téléphone ou internet.

1. Tous les jours ou presque tous les jours
2. Environ une fois ou deux par semaine
3. Toutes les 2-3 semaines
4. Environ une fois par mois
5. Moins qu'une fois par mois
6. Je ne l'avais jamais rencontré(e) précédemment
7. Je préfère ne pas répondre

Lorsque vous avez eu un contact direct avec [NAME] hier, avez-vous eu… ?

1. Un contact physique (n'importe quel type de contact peau contre peau tel que se serrer la main, s'étreindre ou s'embrasser)
2. Un contact non-physique (vous n'avez pas touché cette personne)
3. Je préfère ne pas répondre

Et où avez-vous eu un contact direct avec [NAME] ?
Please tick all that apply

1. Chez moi (y compris à votre porte, dans votre jardin et les accès à votre logement, tels que les escaliers, les ascenseurs et les couloirs)
2. Au domicile de quelqu'un d'autre
3. Au travail
4. Dans un lieu de culte
5. Dans les transports
6. À l'école
7. Dans un magasin de produits de première nécessité, p. ex. un supermarché, un magasin d'alimentation, une pharmacie ou un magasin de vélos
8. Dans un magasin de produits non essentiels, p. ex. un magasin de jardinage, vêtements, produits électroniques, meubles ou bricolage
13. Dans un marché de plein air
9. Dans un lieu de divertissement tel qu'un restaurant, bar, cinéma
10. At a place for sports such as a gym or sports club/match
11. Autre activité dehors (par exemple dans un parc, dans la rue ou à la campagne)
14. Dans un cadre médical, p. ex. hôpital, cabinet d'un médecin, urgences, clinique, dentiste, physiothérapeute, optométriste, etc.
15. Chez un coiffeur, barbier, dans un salon de beauté ou un endroit similaire
12. Ailleurs (veuillez préciser)

Veuillez s'il vous plaît renseigner une approximation du temps passé en face à face avec [NAME] hier.

INSERT HOURS
INSERT MINUTES

L'endroit principal où vous avez rencontré [NAME] hier était-il en plein air ou à l'intérieur ?
Veuillez cocher toutes les réponses qui s'appliquent

1. À l'intérieur
2. À l'extérieur

Nous vous demandons d'inclure tous les contacts que vous avez eus, mais si vous n'avez pas pu tous les inclure (par exemple parce que vous travaillez dans un magasin et avez beaucoup de contacts par jour), pourriez-vous l'indiquer ?

Veuillez sélectionner une seule réponse

1. J'ai inclus toutes les personnes avec qui j'ai eu un contact.
2. Je n'ai pas inclus toutes les personnes avec qui j'ai eu un contact.
3. Je n'ai eu aucun contact

Avec environ combien de personnes non reprises précédemment avez-vous eu contact ? Veuillez donner votre meilleure estimation par âge et endroit.

Pour cette question, nous sommes uniquement intéressés par les personnes que vous avez rencontrées en face à face, ***avec lesquelles vous avez échangé au moins quelques mots, OU avec lesquelles vous avez eu un contact physique****(p. ex. poignée de main, étreinte, sport de contact).*

Veuillez saisir le nombre de contacts pour chaque groupe d'âge et endroit

ROWS
1. Moins de 18 ans
2. Entre 18 et 64 ans
3. 65 ans et plus

COLUMNS
1. Au travail
2. À l'école ou dans un autre environnement d'apprentissage
3. Ailleurs

Q76. Avec environ combien de personnes non reprises précédemment avez-vous eu un contact **physique** ? Veuillez donner votre meilleure estimation par âge et endroit.

Pour cette question, nous sommes uniquement intéressés par les personnes que vous avez rencontrées en face à face ***ET avec lesquelles vous avez eu un contact physique (p. ex. poignée de main, étreinte, sport de contact).***

Veuillez saisir le nombre de contacts pour chaque groupe d'âge et endroit.

ROWS
1. Moins de 18 ans
2. Entre 18 et 64 ans
3. 65 ans et plus

COLUMNS
1. Au travail
2. À l'école ou dans un autre environnement d'apprentissage
3. Ailleurs
