# Supplementary material for: CoMix: comparing mixing patterns in the Belgian population during and after lockdown
Source: Sci Rep. 2020 Dec 14;10:21885. doi: 10.1038/s41598-020-78540-7 (PMC7736856; doi:10.1038/s41598-020-78540-7)
Supplement: Supplementary file 3 — Supplementary Information 3. Ethical approval in Dutch and French [file 41598_2020_78540_MOESM3_ESM.pdf]

MAIN INTRO SCREEN

Merci d’avoir accepté de participer à cette recherche importante à propos de la pandémie du
nouveau
coronavirus (COVID-19). Veuillez s’il vous plait prendre le temps de lire les informations
ci-dessous ainsi
que la notice détaillée de participation au sondage.
Ce sondage est réalisé par Ipsos pour le compte d’un groupe d’experts en modélisation
mathématique
et statistique des maladies infectieuses et en santé publique - London School of Hygiene
and Tropical
Medicine (LSHTM ; Royaume-Uni), l’Université de Hasselt (Belgique), l’Université de
Antwerp (Belgique),
l'Institut National Néerlandais pour la Santé Publique et l'Environnement (RIVM ;
Pays-Bas), l’Université
de Bern (Suisse), et la Fondation ISI (Turin, Italie).
Cette recherche fait partie d'un programme de travail plus vaste financé par la
Commission européenne,
qui vise à fournir les réponses dont nous avons besoin de toute urgence sur les
caractéristiques
épidémiologiques du COVID-19, les dynamiques sociales de l'épidémie, et la
préparation et la réponse
de santé publique à la pandémie en cours, ainsi qu'à évaluer son impact économique.
Elle sera utilisée
directement pour une communication et une interaction active avec les autorités et les
décideurs
politiques, d'autres groupes scientifiques et le grand public, afin de contribuer à
minimiser l'impact du
COVID-19 sur la santé publique, l'économie et la société.
La participation à cette enquête est entièrement volontaire et vous pouvez refuser de la
réaliser.
Toutefois, nous apprécierions vraiment votre soutien car les résultats fourniront des
informations
précieuses pour la prise de décision et l'élaboration de stratégies dans un avenir
proche.

NEW SCREEN

Déclaration de participation
Je confirme avoir 18 ans ou plus.
Je confirme avoir lu et compris les informations relatives à cette étude. J’ai eu l’occasion
d’examiner
ces information avant de participer à l’étude.
Je comprends que la participation à l’étude se fait sur la base du volontariat, et que je suis
libre de
me retirer à tout moment sans donner de raison et sans que mes droits soient affectés.
Je comprends que les sections anonymes collectées durant l’étude pourraient être étudiées
par des
personnes autorisées de l'Université d'Anvers, de l’Université de Hasselt, de la
London School of

Hygiene &; Tropical Medicine, du National Institute for Public Health and Environment aux Pays-
Bas, de l'Université de Berne et de la Fondation ISI de Turin.
Je comprends que des données ANONYMISEES me concernant pourraient être partagées via un
dépôt de données public et que ni moi, ni les membres de mon foyer ne seront identifiables à partir
de ces informations.

Je comprends que ceci est une étude longitudinale, et que je serai invité à remplir plusieurs questionnaires durant l'épidémie en cours.

MAIN INTRO

Bedankt dat u bereid bent om deel te nemen aan dit belangrijk onderzoek over het nieuwe coronavirus (COVID-19). Gelieve even de tijd te nemen om onderstaande informatie en de gedetailleerde nota over deelname aan de enquête aandachtig te lezen.

De enquête wordt uitgevoerd door Ipsos in opdracht van een team van experten op het domein van wiskundige en statistische modellering van infectieziekten en volksgezondheid (de London School of Hygiene & Tropical Medicine (LSHTM; Verenigd Koninkrijk), Universiteit Hasselt (België), Universiteit van Antwerpen (België), Rijksinstituut voor Volksgezondheid en Milieu (RIVM; Nederland), Universiteit van Bern (Zwitserland), en de ISI Foundation in Turijn (Italië).

Het onderzoek is onderdeel van een groter programma dat wordt gefinancierd door de Europese Commissie en is gericht om urgente antwoorden te verkrijgen over de verspreidingskenmerken van het coronavirus, de sociale drijfveren van de epidemie en de gerelateerde paraatheid en respons op het niveau van volksgezondheid ten aanzien van deze epidemie alsook om de economische impact te meten.

Het zal gebruikt worden in actieve communicatie en interactie met beleidsmakers en autoriteiten, andere wetenschappers en de algemene bevolking, om de impact van COVID-19 op de volksgezondheid, economie en maatschappij te minimaliseren.

Deelname aan deze enquête is volledig vrijwillig en u kan weigeren om deel te nemen. We zouden uw hulp echter enorm waarderen omdat de resultaten zeer belangrijke informatie opleveren ter ondersteuning van het nemen van beslissingen en het ontwikkelen van strategieën in de nabije toekomst.

ETHICS FORM FOR PARTICIPANTS

Deelname Verklaring

Ik ben 18 jaar of ouder.

Ik bevestig dat ik de informatie over dit onderzoek heb gelezen en begrepen. Ik heb de kans gehad om hierover na te denken alvorens mijn deelname aan deze studie te bevestigen.

Ik begrijp dat mijn deelname aan dit onderzoek volledig vrijwillig is, en dat ik op elk moment zonder opgaaf van reden vrij ben om met dit onderzoek te stoppen. Indien ik met het onderzoek besluit te stoppen, word ik daarmee niet in mijn rechten aangetast.

Ik begrijp dat bevoegde personen van de Universiteit van Antwerpen (België), Universiteit van Hasselt (België), London School of Hygiene & Tropical Medicine (Verenigd Koninkrijk), Rijksinstituut voor Volksgezondheid en Milieu (RIVM; Nederland), Universiteit van Bern (Zwitserland), en de ISI Foundation Turijn (Italië) bepaalde gedeeltes van mijn geanonimiseerde data kunnen inzien.

Ik begrijp dat GEANONIMISEERDE data over mij via een publiek toegankelijke databank gedeeld kunnen worden, en dat ik niet door deze data geïdentificeerd kan worden.

Ik begrijp dat dit onderzoek gedurende meerdere weken herhaald zal worden, en dat ik voor meerdere vragenlijsten gedurende de Covid-19 epidemie uitgenodigd zal worden.

Indien u meer informatie wenst over deze studie kan u de IPSOS helpdesk contacteren (epanel-be@i-say.com).
